# An 8-Week, Low Carbohydrate, High Fat, Ketogenic Diet Enhanced Exhaustive Exercise Capacity in Mice Part 2: Effect on Fatigue Recovery, Post-Exercise Biomarkers and Anti-Oxidation Capacity

**DOI:** 10.3390/nu10101339

**Published:** 2018-09-20

**Authors:** Qingyi Huang, Sihui Ma, Takaki Tominaga, Katsuhiko Suzuki, Chunhong Liu

**Affiliations:** 1 College of Food Science, South China Agricultural University, Guangzhou 510642, China; hqyaaaaaa@163.com; 2 Graduate School of Sport Sciences, Waseda University, Tokorozawa 359-1192, Japan; masihui@toki.waseda.jp (S.M.); t.tominaga7713@gmail.com (T.T.); 3 The Key Laboratory of Food Quality and Safety of Guangdong Province, Guangzhou 510642, China; 4 Faculty of Sport Sciences, Waseda University, Tokorozawa 359-1192, Japan

**Keywords:** ketogenic diet, keto-adaptation, fatigue recovery, oxidative stress

## Abstract

A low-carbohydrate, high-fat ketogenic diet (KD) is a nutritional approach ensuring that the body utilizes lipids. In our previous study, we found that an eight-week ketogenic high-fat, low-carbohydrate diet increased the capacity of endurance exercise in mice without aggravated muscle injury, despite the decrease of absolute muscle volume. The potential mechanism is most possibly to be enhanced capacity to mobilize and utilize fat. In the present study, we investigated whether a ketogenic diet influences post-exercise recovery by measuring blood biomarkers, muscle and liver oxidative state as well as fatigue recovery 24 h post exercise by employing an open-field locomotion test. Several biochemistry markers indicating exercise-induced injury after exhaustive exercise were improved by KD, followed by a 24-h rest with free feed access, including lactate. No aggravated hepatic oxidative damage was observed, whereas muscular oxidative stress was increased by KD. Accelerated recovery induced by exhaustive exercise was also observed from blood biomarkers of injury. For fatigue recovery, lactate concentration, a marker often employed as exhaustion index was lowered by KD, whereas an open field test showed that KD application contributed to increased locomotion after exhaustive exercise, followed by a 24-h rest. These results suggest that KD has the potential to be used as a fatigue-preventing and/or recovery-promoting diet approach in endurance athletes.

## 1. Introduction

Marathons and prolonged endurance exercises are usually accompanied by fatigue and tissue/organ damage, e.g., muscle damage, acute kidney injury and hepatic dysfunction [1,2,3]. Several markers are employed for exercise-induced tissue/organ damage, such as aspartate transaminase (AST), alanine transaminase (ALT) and gamma-glutamyl transpeptidase (γ-GTP) for hepatic damage, creatine kinase (CK) and lactate dehydrogenase (LDH) for muscle damage, blood urea nitrogen (BUN) and creatinine for renal damage, and amylase and lipase for pancreas permeability [4,5,6,7,8]. To counter the negative impacts of endurance exercise-induced injuries, nutritional approaches, such as sports supplements, and macro- or micronutrients administration, are often adopted [5,9,10,11].

Fatigue means inability of continuous exercise, inducing secondary exercise intolerance. Accumulation of different metabolites, such as lactate, Pi, ammonia or Ca^2+^, the depletion of glycogen, which is the main energy origin in a carbohydrate-centered metabolic system, and oxidative stress during endurance exercise may result in fatigue [12,13,14,15,16]. Thus, plasma lactate level was investigated in the present study.

A low-carbohydrate, high-fat ketogenic diet (KD) is a nutritional approach ensuring that the body utilizes lipids. In our previous study, an 8-week ketogenic high-fat, low-carbohydrate diet increased the capacity of endurance exercise in a mouse model, without aggravated muscle injury, despite the decrease of absolute muscle volume [10]. To investigate the post-exercise plasma biochemistry and organ damage, we aim to analyze plasma metabolite concentrations as well as biomarkers of tissue/organ damage.

Reactive oxygen species (ROS) are derived from molecular oxygen, containing a number of reactive molecules and free radicals. ROS have long been known for its defense response against microbial invasion, functioning as a weapon of immune cells during inflammation. Though beneficial for skeletal muscle regeneration at an appropriate level, exhaustive exercise, especially repetitive muscle contraction, may cause oxidative stress [17,18,19]. Micronutrients such as antioxidants have been reported for improving endurance capacity, and such supplements are being applied in endurance competitive events [20,21]. Moreover, oxidative stress is also reported to be associated with exercise-induced fatigue [22,23]. In the present study, we investigated whether KD influenced the muscle and liver oxidative stress state, by observing tissue-located lipid and protein oxidation after exhaustive exercise. 

Mice and many other rodents are genetically decided to adopt continuous locomotion, moving or running around [24,25]. Locomotion is suspected to decrease when achieving partial or absolute fatigue, and there are reported studies using open-field tests to evaluate fatigue level [26]. It is hard for us to distinguish between physical exhaustion and volitional exhaustion in human trials. While exploiting the nature of rodents, decreased movement may be a way to determine fatigue, or to measure recovery. To investigate whether KD could affect fatigue recovery, we enriched this study with an open-field test immediately or 24 h after exhaustive exercise.

## 2. Materials and Methods

### 2.1. Mouse Maintenance and Diets

Male C57BL/6J mice (*n* = 35) were purchased from Takasugi Experimental Animals Supply (Kasukabe, Japan) at 7 weeks of age. Four or five animals were housed together in 1 cage (27 × 17 × 13 cm) in a controlled environment under a light–dark cycle (lights on at 0800 and off at 2000). The experimental procedures followed the Guiding Principles for the Care and Use of Animals in the Academic Research Ethical Review Committee of Waseda University and were approved (10K001). All mice were randomly divided into four groups: chow diet (control: Con), including chow diet, sedentary (*n* = 8) and chow diet plus exercise (Con + Ex, *n* = 9), ketogenic diet (KD), including KD, sedentary (*n* = 9) and KD plus exercise (KD + Ex, *n* = 9) groups. A KD diet TP-201450 (consisting of 76.1% fat, 8.9% protein and 3.5% carbohydrate, 7.342 kcal/g) and a chow diet AIN93G (consisting of 7% fat, 17.8% protein and 64.3% carbohydrate, 3.601 kcal/g) wt/wt were obtained from TROPHIC Animal Feed High-tech Co., Ltd. (Nantong, China). The mice were kept on ad libitum chow diet or KD. 

### 2.2. Endurance Capacity Test Protocol

One week before exhaustive exercise, all mice were accustomed to treadmill running at 15 m/min for 10 min. The endurance test was performed on a motorized treadmill (Natsume, Kyoto, Japan). That is, mice in the Con + Ex and KD + Ex groups were subjected to treadmill running at 10 m/min for 15 min, followed by 15 m/min and 20 m/min for 15 min each, and then 24 m/min and 7% grade until exhaustion. The protocol was approved by the Academic Research Ethical Review Committee. Exhaustion was defined as the inability to continue regular treadmill running despite the stimulation of repeated tapping on the back of the mouse. The running time of the mice was recorded. Immediately or 24 h after the exhaustion, the mice were terminated under light anesthesia with inhalant isoflurane (Abbott, Tokyo, Japan). Blood samples were taken using heparin from the abdominal aorta under inhalant isoflurane-induced mild anesthesia, and tissues and organs were immediately excised and frozen in liquid nitrogen. Plasma was obtained from blood samples by centrifugation at 1500 g for 10 min at 4 °C. These samples were stored at −80 °C until analyses.

### 2.3. Open Field Analysis

The chamber used for this test was 50 cm (length) × 50 cm (width) × 38 cm (height) and was made of white high-density and non-porous plastic. 95% Ethanol was used to wipe the chamber each time prior to use and before subsequent tests to remove any scent clues left by the previous subject mouse. Each subject mouse was tracked for a single 10 min free and uninterrupted period, during which time the tracking software recorded movement. Total travelling time was recorded and analyzed using a digital camera [24,25,26].

### 2.4. Plasma Biochemical Assessment

Plasma levels of glucose, non-esterified fatty acids (NEFA), triglyceride (TG), lipase, aspartate transaminase (AST), alanine transaminase (ALT), creatine kinase (CK), lactate dehydrogenase (LDH), blood urea nitrogen (BUN), cholesterol (CHO), high-density lipoprotein cholesterol (HDL), low-density lipoprotein cholesterol (LDL) and albumin were measured by Koutou-Biken Co. (Tsukuba, Japan). 

### 2.5. Lactate Assay

Plasma lactate was measured using an EnzyChrom Ketone Body Assay Kit (Bio Assay Systems, Hayward, CA, USA) according to the manufacturer’s instructions. Plantaris muscle lactate was measured using the Lactate Microplate Assay Kit (Laibio Co., Shanghai, China).

### 2.6. Measurement of Oxidative Stress

For plantaris muscle and liver oxidative stress evaluation, 1 mg of each tissue (wet weight) was homogenized with PBS, after which the Thiobarbituric Acid Reactive Substances (TBARS) Microplate Assay Kit and the Protein Carbonyl Assay Kit (Laibio Co., Shanghai, China) were employed and assays were performed according to the manufacturer’s instructions.

### 2.7. Statistical Analysis

Data were presented as means ± standard deviations (SD). A two-way analysis of variance (ANOVA) was performed to determine the main effects of diet and/or exercise. Statistical analysis was done using Graphpad 7.0 (Graphpad, Ltd., La Jolla, CA, USA). When this analysis revealed significant interaction, Tukey’s post-hoc test was performed to determine the significance among the means. Statistical significance was defined as *p* < 0.05.

## 3. Results and Discussion

### 3.1. KD Lowered Mice Weight 

**Figure 1:**
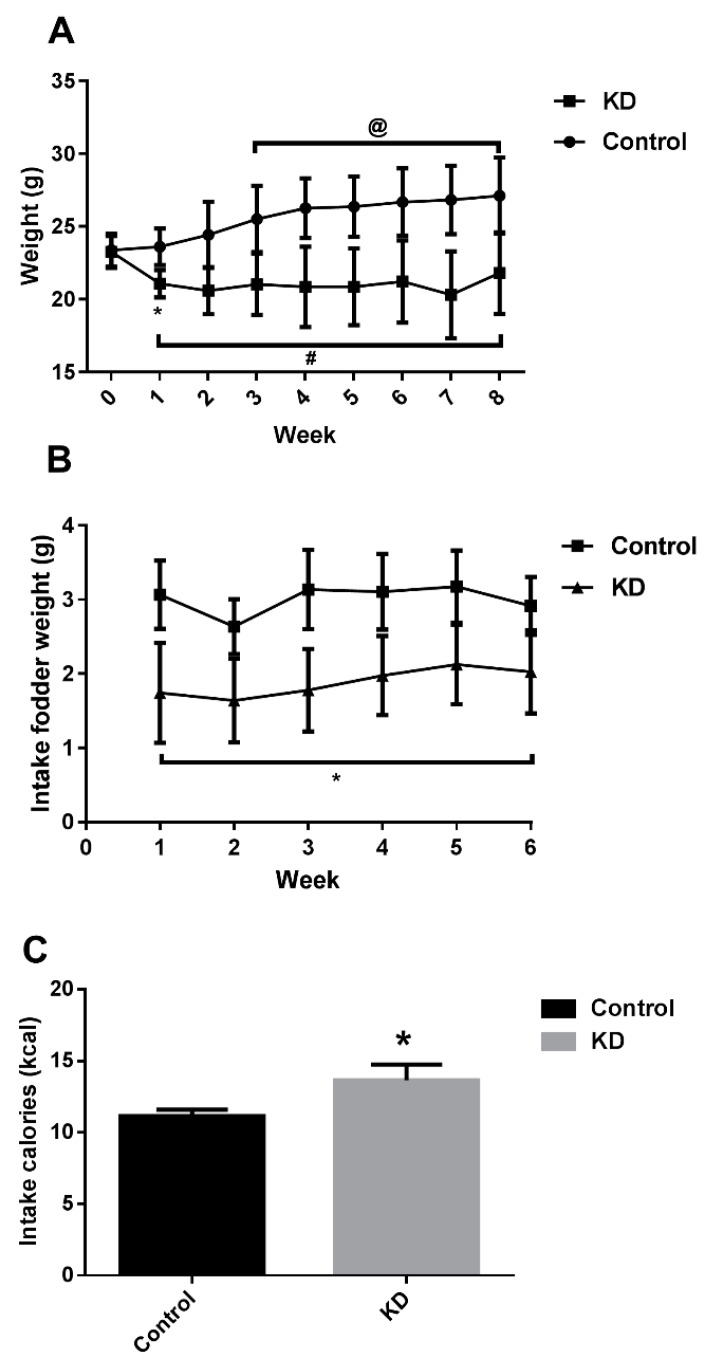
Effects of different diet feeding on weight and energy intake. (**A**) 8-week of ketogenic diet (KD) feeding led to significant weight loss. * *p* < 0.01, compared with KD week 1. @ *p* < 0.01 compared with chow week 1. # *p* < 0.01 compared with chow of the same week. (**B**) Food intake (daily, individually) variation throughout the experimental period. * *p* < 0.01, compared with chow of the same week. (**C**) Caloric intake was greater in KD-feeding mice than in chow diet-feeding mice. * *p* < 0.001 vs. chow.

We have reported that although mice in the KD group consumed more calories than the control group, they showed significant weight loss after 1-week of KD administration [10]. Figure 1 shows different nutrition division results in body weight and composition. The potential of KD for weight loss has been widely reported worldwide over decades. The short-term ability of a well-designed KD for weight loss is prominent in a variety of experimental subject models, including humans, rodents and aquatics [10,27,28]. However, the long-term effect of KD on weight control is yet to be concluded, since several studies have reported disappointing results. Studies on over 1-year KD administration has yielded low-efficacy compared to low-fat diet administration [29,30]. The reasons for this phenomenon may be appetite influence and adapted metabolism [29,30,31].

Obesity is especially troublesome; though the adoption of exercise as a therapy is effective, the exercise capacity drops in obese subjects, accompanied with a decreased desire to participate in sports. Reversely, the less patients want to exercise, the weaker their exercise capacity, including cardiorespiratory function and muscle strength, which may drop more severely. In the FIT (Henry Ford Exercise Testing) project carried out recently, researchers assessed the association of body mass index (BMI) and exercise capacity with all-cause mortality using multivariable-adjusted Cox proportional hazards models, claiming that exercise capacity-obesity paradox dichotomy exists [32].

Our previous study showed that a 2-month KD administration enhanced endurance exercise capacity without aggravated muscle damage, indicating that KD combined with exercise therapy may be a better medicine for obese subjects, whereas exercise training may be a better prevention for weight rebound caused by long-term KD. 

### 3.2. KD Accelerated Fatigue Recovery after a 24-h Rest

**Figure 2:**
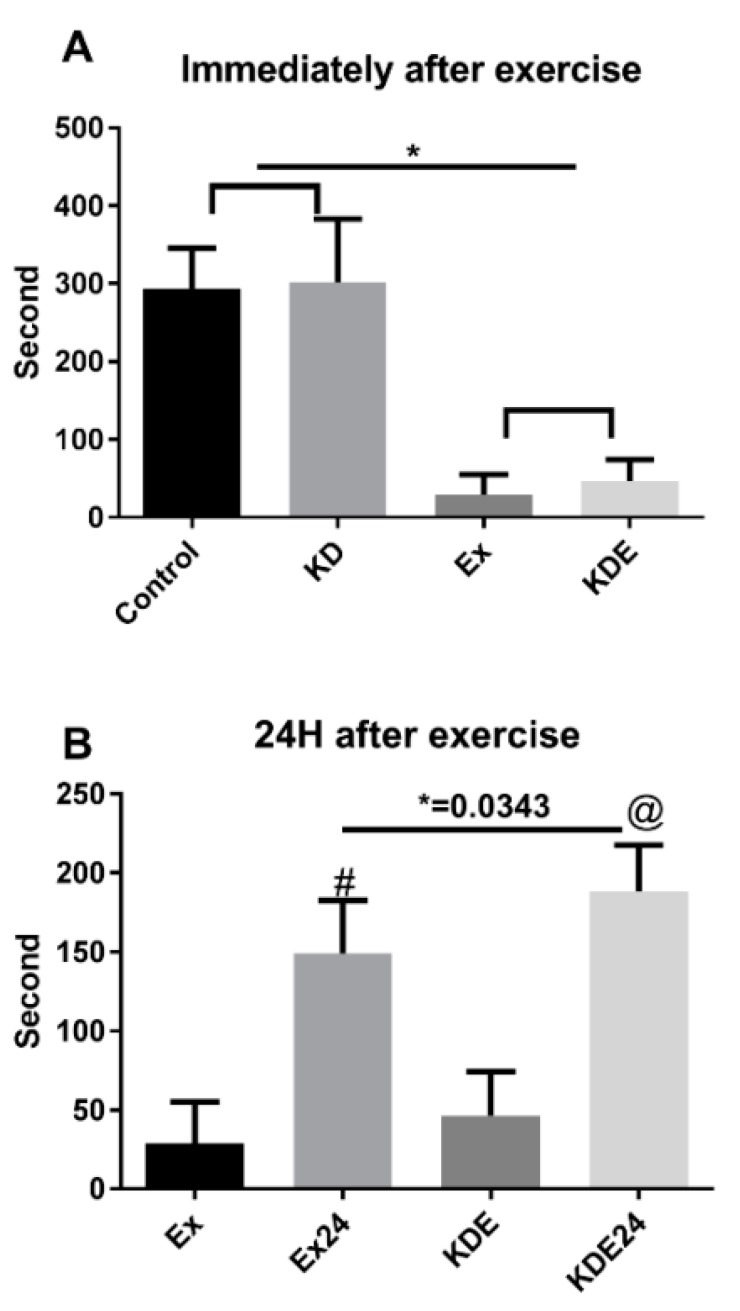
Locomotion time of mice (**A**) immediately or (**B**) 24 h after exhaustion as indicated. * *p* < 0.05 compared with indicating column, @ *p* < 0.05 compared with KDE, # *p* < 0.05 compared with Ex.

**Figure 3:**
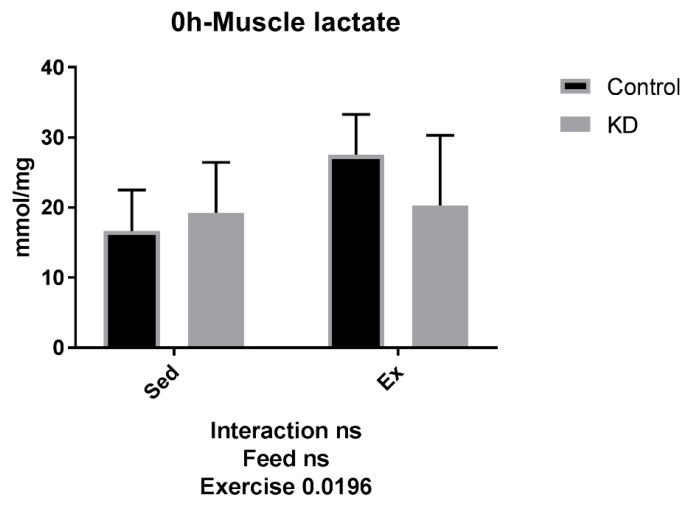

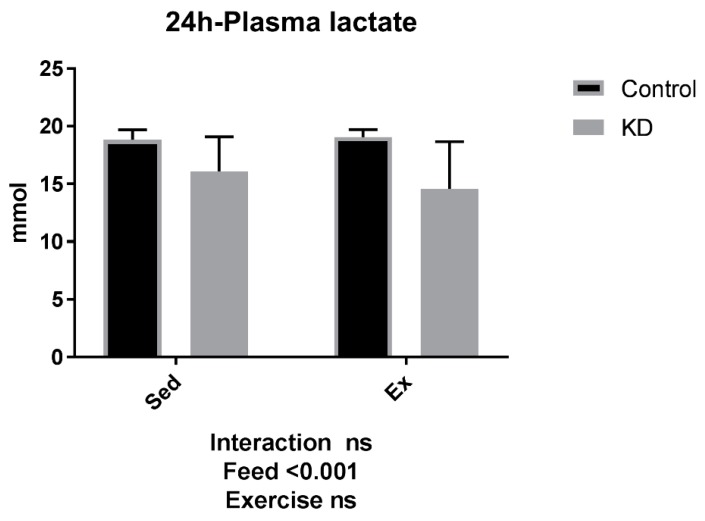
Muscle and plasma lactate concentration of mice 24 h after exhaustion.

Open field tests are effective methods to investigate locomotor behaviors [33]. In the present study, an open field test was employed as a variable for exercise-induced fatigue level. As shown in Figure 2A, due to exhaustion, subjects in both groups almost stayed right in the same place where it was laid. However, after the 24-h recovery, KD-feeding mice showed an accelerated recovery phase. As shown in Figure 2B, after a 24-h rest, KD mice moved around more frequently compared to Con mice. To examine the mechanism, we performed a lactate assay in muscle tissue. As shown in Figure 3, though exhaustive exercise induced lactate accumulation in plantaris muscle of Con mice after exercise, it did not induce significant lactate accumulation in counterparts of KD. Moreover, the baseline of lactate acid is significantly lower for KD subjects compared to Con subjects. In the early stage study of KD on exercise performance, well-trained athletes were usually the subjects. A classical study was conducted in highly-trained endurance athletes, to test how KD affects submaximal exercise capacity and metabolites levels. At exhaustion, substrate dynamics showed that post-exercise lactate level was decreased after a 4-week eucaloric KD attribution (2.77 ± 0.61 vs. 2.41 ± 0.27, mmol/L) [34]. This was confirmed in the present study, using rodent models. Exercise-induced fatigue could be attributed to peripheral fatigue at the neuromuscular junction, and central fatigue within the brain through the muscle-nerve axis [35]. During exercise, most lactate is produced in the skeletal muscle. There was a significant negative correlation between aerobic endurance performance and blood lactate value five min after exercise [36]. Well-trained endurance runners have more effective lactate-producing ability and higher lactate clearance rate than non-trained individuals [37]. After exercise, lactate is mainly oxidized into CO_2_ and water in skeletal muscle and myocardium. The transfer process of the lactate shuttle can include ingredients of synthesis of hepatic glycogen or biosynthesis of fatty acids and certain amino acids. Premium lactate clearance capacity is beneficial to the removal of lactate, thus promoting recovery after exercise. Several strategies, such as rest, sports massage and active recovery, were, thus, attempted to find effective ways to clear lactate more quickly [38,39]. Among them, sports massage treatment showed beneficial effects on lactate clearance [38].

Another metabolite that is accumulated during exercise is blood ammonia, a classical biochemical marker for exercise-induced fatigue [40]. Exercise may cause uremia, a condition of having increased blood ammonia levels, reported in rats and humans after exhausting exercise [41]. During fatigue, ammonia may enter across the blood brain barrier, and cause central fatigue. Ammonia level was reported to be correlated with BUN (*R*^2^ = 0.95), the final product of blood ammonia. In fact, we observed a decrease of BUN in KD group, and this may be a consequence of restricted protein content in KD, that may lead to low blood ammonia level, thus contributing to a low accumulation level in the brain, and quick fatigue recovery. In summary, exercise-induced fatigue is reported to be related with lactate and ammonia accumulation. It is reported that cluster multi-diode light therapy could cause a tiny delay in skeletal muscle fatigue, decrease post-exercise blood lactate levels and inhibit the release of CK and C-reactive protein. The ability for KD to accelerate fatigue recovery may be accredited to keto-adaptation, a system that differs from adapted glycogen utilization or glycolysis, where most lactate may come from. Moreover, the properties of KD to accelerate lactate clearance may favor the all-cause mortality in trauma patients, since it has been validated for its efficacy in a meta-analysis [42]. 

Results from this part indicated that KD might be promising to be used as a post-competition diet to boost recovery. Excessive oxidation is also reported to be related with exercise-induced fatigue. To understand more about the mechanism, we analyzed post-exercise biochemistry and tissue-specific oxidative stress.

### 3.3. Plasma Substrate Concentrations Were Altered by KD after a 24-h rest

KD-feeding mice exhibited an elongated endurance capacity than chow-feeding mice [10]. Though glucose was significantly decreased by exhaustive exercise in the chow-feeding group, it was recovered after a 24-h rest. The baseline of glucose was significantly lower in the KD group, and this might be attributed to the low coefficient of glucose utilization in the KD mice, since they mainly maintained a supply system strongly powered by fat oxidation.

**Figure 4:**
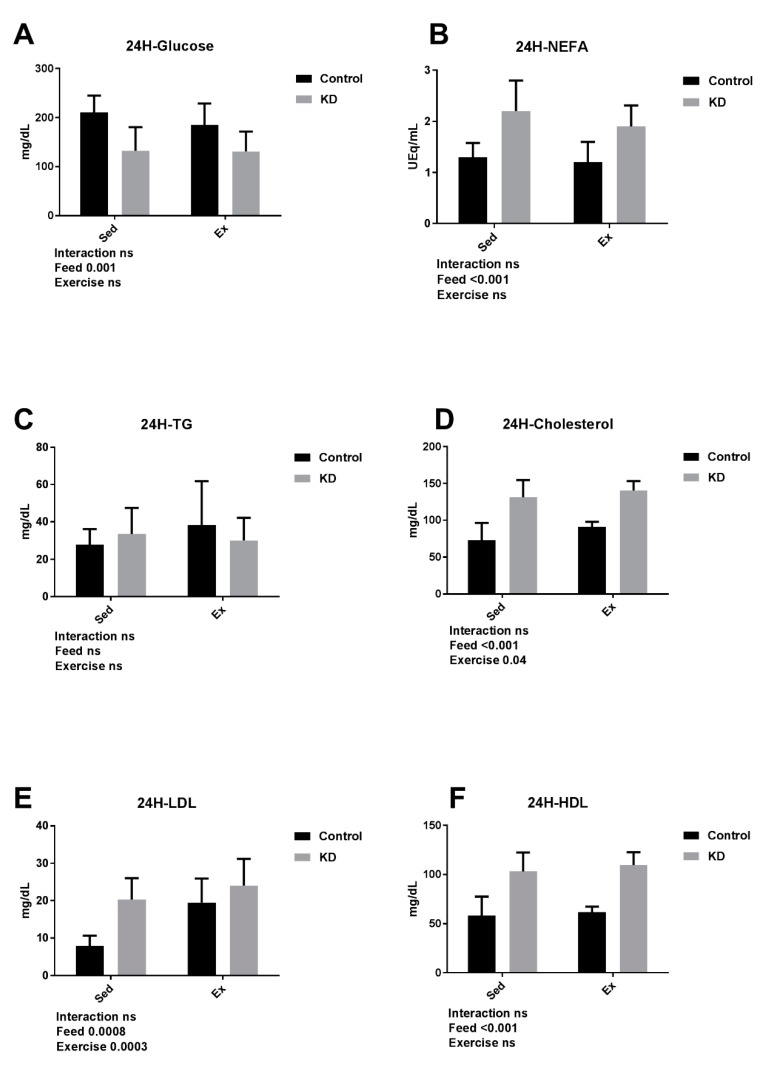
Plasma biochemistry results 24 h after exhaustion as indicated. (**A**–**F**) Concentrations of plasma glucose, non-esterified fatty acids (NEFA), triglyceride (TG), cholesterol (CHO), high-density lipoprotein cholesterol (HDL) and low-density lipoprotein cholesterol (LDL).

Immediately after strenuous exercise, subjects are inclined to achieve a hyperlipidemia status [10,43]. As shown in Figure 4, in the baseline, NEFA concentration was elevated by KD feeding, which was also confirmed by other studies. In the study, discussed in Section 3.2, at exhaustion, NEFA level was increased by KD, compared to an eucaloric balanced diet (1.25 ± 0.13 vs. 1.60 ± 0.31, mmol/L). In our study, no difference was observed between sedentary or exercise mice after a 24-h rest after exercise. Only feeding contributed to the significance between the groups. According to previous study, KD contributed to increased NEFA utilization during strenuous exercise, whereas after a 24-h rest, plasma NEFA concentration rebounded quickly. This result indicated enhanced metabolic flexibility in keto-adapted mice. At 24 h after exercise, the concentration of TG did not differ from each group, although immediately after exercise, TG concentration decreased significantly in KD mice. These results combined together showed that 24 h after exhaustive exercise, neither NEFA nor TG was altered compared to the same-fed groups. 

LDL and HDL are components of CHO. As shown in Figure 4, CHO, LDL and HDL were significantly elevated by KD, which was consistent with our earlier results. Our results showed that the concentrations of CHO, LDL and HDL were all elevated after a 24-h rest after exercise, although they decreased immediately after exercise. Several studies reported that KD resulted in high concentration of blood CHO, whereas exhaustive exercise reduced the concentration of CHO and lipid profile [44,45,46,47]. Our results showed that KD was involved in the elevation of either CHO or its component LDL and HDL. Moreover, after a 24-h rest, once-reduced plasma LDL, HDL and CHO experienced a rebound, indicating that an “overcompensation” does not only exist in muscular strength, but also in plasma substrates.

### 3.4. KD Contributed to an Accelarated Damage Recovery 

**Figure 5:**
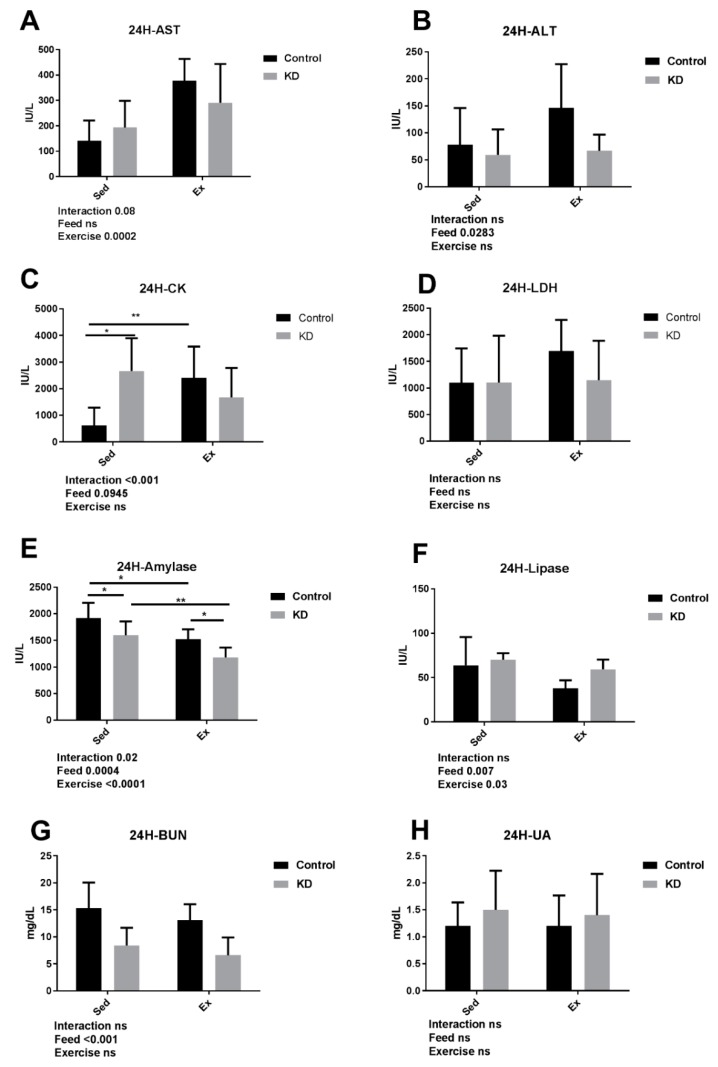
Plasma organ damage markers 24 h after exhaustion. (**A**–**H**) Concentrations of plasma aspartate transaminase (AST), alanine transaminase (ALT), creatine kinase (CK), lactate dehydrogenase (LDH), amylase, lipase, blood urea nitrogen (BUN) and urea acid (UA). * *p* < 0.05, ** *p* < 0.01.

As shown in Figure 5, AST and ALT, employed as hepatic damage markers, still remained in higher concentration in the chow-feeding group after a 24-h rest. Immediately after exercise, ALT was significantly lowered by KD, showing a preventive effect towards hepatic damage caused by exhaustive exercise [10]. Interestingly, this situation is different in the KD group, indicating that KD may boost hepatic damage recovery. In our study, KD did not aggravate hepatic damage caused by exhaustive exercise, which is different from previous studies [48,49,50]. Hepatic damage produced by exercise is highly presumed to be related to free radicals [51]. In fact, a two- to three-fold increase in free radical concentrations of liver at exhaustion was reported [52]. To further study the mechanism of this protection, we investigated the oxidative stress markers of the liver, which is discussed later.

CK and LDH are employed as muscle damage markers. LDH exhibited no significant difference in each group. However, CK was elevated by exercise even after a 24-h rest, and the situation was different in the KD group. In our previous study, KD did not alter immediate post-exercise CK or LDH concentration; the damage was not made worse by KD-induced muscle weight loss [10]. However, KD lowered both CK and LDH (no significance observed) after a 24-h rest, indicating that KD managed to protect the muscle from delayed damage. Muscles experience damage and inflammation during recovery from exercise, causing delayed-onset muscle soreness (DOMS). Several methods were attempted to relieve this phenomenon, such as foam rolling and supplementation [53,54]. However, the practicability of these findings of KD in human athletes needs to be investigated further.

Amylase was decreased by KD or a 24-h rest after exercise, which is the opposite of the results gathered immediately after exercise [10]. The increase in plasma amylase immediately after exercise may be a consequence of aggravated pancreas permeability caused by exercise; a knock-on effect may be caused afterwards [55,56,57]. A combination of KD feeding, restricted carbohydrate supply, and abundant fat supply cause keto-adaptation, and a weakened glucose-powered energy supply system. The weakened role of amylase may result in its low concentration in the blood. A delayed glucose-metabolism reduction was observed here. Similarly, lipase was decreased by exercise in the chow group, which exhibits the same pattern as amylase. This may be attributed to a delayed recovery effect of the body.

As discussed in Section 3.2, BUN is employed as a kidney injury marker, as well as an indicator of exercise tolerance. At the same time, it is also a protein degradation marker. BUN was significantly lower in KD groups after 24-h post exercise compared to the chow group. This may be partly attributed to the lower protein content in KD, indicating that it may possess a protective ability towards renal damage caused by exhaustive exercise. However, it is reported that a high-fat diet may induce renal oxidative damage via the ketogenesis/ sirtuin(SIRT)3 pathway, indicating that KD may lead to renal dysfunction [8,58]. Administration of antioxidants seems to be an applicable method to ameliorate this adverse effect [59].

Strenuous exercise has also been well documented as a cause of increase in the number of circulating immune cells, including neutrophils and monocytes, which are recruited from circulating blood to local tissues, thus inducing a systemic inflammatory response [1]. Exercise-triggered inflammatory cytokines, including growth hormone, interleukin (IL)-1beta, IL-6, tumor necrosis factor alpha and interferon gamma, may induce systemic or local inflammatory responses, while mobilized neutrophils or monocytes may infiltrate muscle or other organs, thus causing local inflammation, leading to further recruitment of immune cells into tissues, eliciting cascade amplification of inflammation and organ failure [1,2,3]. It is also reported that exercise-induced IL-1beta may lead to central nerve system fatigue [60]. Taking these findings into consideration, we find that KD may boost fatigue recovery through a low level of circulating inflammatory cytokines. Further studies should pay attention to this issue. In summary, KD interacts with blood biomarker alterations 24-h post exercise. However, further investigations should be made on the potential side-effects of KD attribution and its association with inflammation. 

### 3.5. TBARs and Protein Carbonyl Level Is Not Alternated by KD

Oxidative stress during exhaustive exercise shows an imbalance between pro-oxidant/anti-oxidant homeostasis. ROS exhibits extremely short half-lives; however, there are several ways to measure ROS activity indirectly, by measuring endogenous anti-oxidative ability, oxidized products of biomolecules attacked by ROS [17,18,19]. TBARS are formed as a byproduct of lipid peroxidation, whereas protein carbonyl groups are products of specific protein side chains oxidized by ROS attacks [61,62]. Exhaustive exercise is reported to increase oxidative stress in various organs and tissues; in this context, a nutritional approach or nutrient supplementation that may reduce abnormal oxidative stress in such organs can enhance exercise performance and contribute to post-exercise recovery [20,63,64].

**Figure 6:**
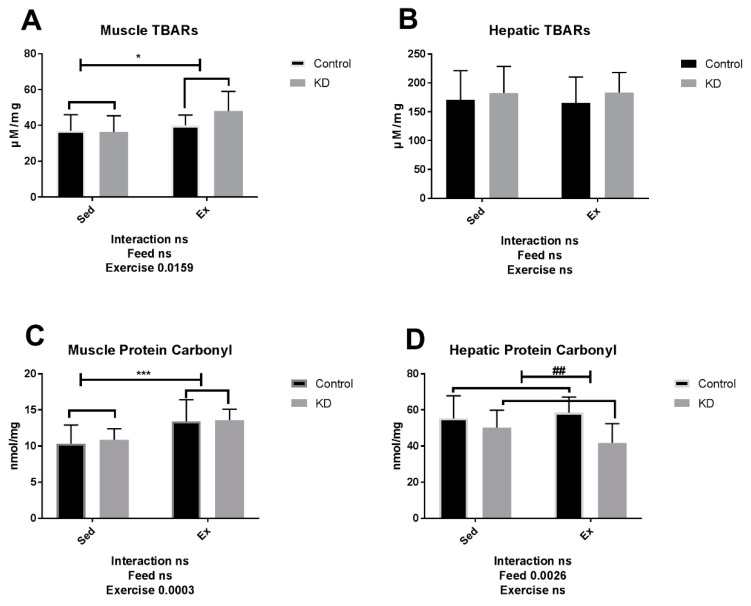
Muscle and hepatic oxidative stress immediately after exhaustion. (**A,B**) TBARs in plantaris muscle tissue and liver tissue. (**C,D**) Protein carbonyl in plantaris muscle tissue and liver tissue. * *p* < 0.05, *** *p* < 0.001 compared between sedentary and exercise, ## *p* < 0.01 compared between control diet and KD.

Discussion about the anti-oxidative properties of KD were controversial according to previous reports [65,66,67,68]. As shown in Figure 6A,C, muscle TBARs and protein carbonyl levels were both significantly enhanced by acute exercise. Though a lipid-enriched KD did not drive rapid increase of lipid oxidation in the sedentary stage, it contributed, on some level, to exercise-induced oxidative stress. As shown in Figure 6B,D, it seems that neither lipid peroxidation nor protein side chain oxidation has deteriorated by exhaustive exercise or KD feeding in muscle tissue; however, protein side chain oxidation was attenuated by KD, and this may partly explain how KD protects the liver from exercise-induced damage, since exercise-induced free radicals were reported to induce liver injuries [51,52]. In summary, oxidative stress was only partially reduced by KD administration. Our study did not observe meaningful regulation by KD in the format of lipid oxidation or protein side chain oxidation measurement. The results here might partly be a result of the low-protein and high-fat content of the different fodder. However, it is hard to define whether the aggravation of ROS is beneficial or not, since at low concentration, ROS is beneficial for muscle repair, while at relative high levels, it may be harmful for muscle health [69]. The curve of this beneficial/harmful effect is bell-shaped. It has been confirmed that exhaustive exercise causes aggravated oxidative damage. When implementing a high-fat diet in athletes, it is necessary that their oxidative state is occasionally monitored; an antioxidant application is also recommended. 

The results of experiments on rodent models may not completely translate to humans, primarily because we share different metabolic systems, e.g., human and rodents have dependent species-specific glucose metabolism; for primates, the skeletal muscle tissue is the primary site of glucose utilization, but for rodents, it is the liver. Studies on caloric restriction (CR) diets (like KD) are a good example of research methods that employ various experiment models. The anti-ageing effect of CR is reproducible in various species, including nematoda, rodent, and primates [70,71]. KD has been clinically used for decades as a treatment for neurologic illnesses (e.g., epilepsy and traumatic brain injury), metabolic illnesses (e.g., glucose transporter type 1 deficiency and McArdle disease) and cancer. It has also been studied for its function mechanism, usually in rodent models, and the results are reproducible in the species on individual levels [72,73,74]. Thus, we believe that the results here have potential to be successfully translated into humans though we share a heterologous genome. However, the results of an animal study may have limitations due to the difference in protein structure encoded by different nucleic acids, cellular communication, and transcriptional regulation between species. The clinical efficacy of KD for fatigue recovery should be further validated.

## 4. Conclusions

In the present study, we investigated the impact of an eight-week keto diet (KD) on post-exercise protection of muscle and organ damage, as well as the influence of the diet on fatigue recovery. Along with enhanced exercise performance, the muscle damage caused by exhaustive exercise was attenuated by KD. The diet also enhanced fatigue recovery after exhaustive exercise. However, it did not have a regulatory effect on muscle lipid oxidation or protein side chain oxidation. These results suggest that KD has the potential to be used as a fatigue-preventing and/or recovery-promoting diet approach for endurance athletes (their oxidative state should be monitored from time to time).
